# Putting the available chemical space to the fingertips of our scientists

**DOI:** 10.1186/1758-2946-4-S1-O7

**Published:** 2012-05-01

**Authors:** Jörg Degen, Mark Rogers-Evans, Daniel Stoffler

**Affiliations:** 1 Cheminformatics & Statistics, F. Hoffmann-La Roche Ltd., Basel, Switzerland; 2 Discovery Chemistry, F. Hoffmann-La Roche Ltd., Basel, Switzerland

## 

One of the most important and frequently occurring questions to answer in the course of a drug design project is which compounds to synthesize next. If it were possible to search virtually all available molecules via one interface, new chemical design suggestions and ideas could be followed up more quickly, thus drastically reducing the cycle time needed for hypotheses testing. Furthermore, if there was a platform available that offered sufficient flexibility for combining various constraints and for handling fuzziness in chemical substructures, promising and non-obvious chemical variations could be identified more quickly and easily, resulting in a more efficient exploration of chemical space.

As a result of a close and long standing collaboration between Discovery Chemistry and Cheminformatics, we have established a global system that allows every researcher at Roche to access all available small molecules that can either be obtained from internal sources or via external vendors. For the more than 10 Million physically accessible compounds,, the associated structural and physicochemical properties are included as well as information on existing quantities, locations, prices and availability. Our tailored designed web-based interface and underlying query engine allows for the identification of compounds using various constraints and simultaneously provides utmost flexibility for combining different search criteria. Similarity and particularly fuzzy search capabilities facilitate an intuitive navigation of the available chemical space. The whole system is very fast so that query results are returned almost instantly, allowing for a quick and interactive drill-down to manageable sets of compounds.

The system is being used globally by a diverse user community and applied for addressing many different kinds of questions, for example, for fast identification of hit expansion candidates, exploration of building block libraries, probing of our in-house screening library, compilation of screening compound subsets, or finding appropriate scaffold replacements. To our knowledge, there is no other platform available that provides similar flexibility, performance and usability for accessing diverse chemicals at such speed.

During the presentation we will outline the basic concepts and architecture of the system and point out some key challenges that we were facing during its development. In addition, we will give some examples on how we integrated and search for recent Roche-ETH driven developments of potent, novel, alternative building blocks in medicinal chemistry [1].
